# FSH Requirements for Follicle Growth During Controlled Ovarian Stimulation

**DOI:** 10.3389/fendo.2019.00579

**Published:** 2019-08-27

**Authors:** Ali Abbara, Aaran Patel, Tia Hunjan, Sophie A. Clarke, Germaine Chia, Pei Chia Eng, Maria Phylactou, Alexander N. Comninos, Stuart Lavery, Geoffrey H. Trew, Rehan Salim, Raj S. Rai, Tom W. Kelsey, Waljit S. Dhillo

**Affiliations:** ^1^Section of Endocrinology and Investigative Medicine, Imperial College London, Hammersmith Hospital, London, United Kingdom; ^2^IVF Unit, Hammersmith Hospital, Imperial College Healthcare NHS Trust, London, United Kingdom; ^3^School of Computer Science, University of St Andrews, St Andrews, United Kingdom

**Keywords:** ovarian response, follicle growth, recombinant FSH, *in vitro* fertilization (IVF), reproduction, fertility

## Abstract

**Introduction:** Ovarian follicle growth is a key step in the success of assisted reproductive treatment, but limited data exists to directly relate follicle growth to recombinant FSH (rFSH) dose. In this study, we aim to evaluate FSH requirements for follicular growth during controlled ovarian stimulation.

**Method:** Single center retrospective cohort study of 1,034 IVF cycles conducted between January 2012–January 2016 at Hammersmith Hospital IVF unit, London, UK. Median follicle size after 5 days of stimulation with rFSH and the proportion of antral follicles recruited were analyzed in women treated with rFSH alone to induce follicular growth during IVF treatment.

**Results:** Starting rFSH dose adjusted for body weight (iU/kg) predicted serum FSH level after 5 days of rFSH (*r*^2^ = 0.352, *p* < 0.0001), median follicle size after 5 days of rFSH, and the proportion of antral follicles recruited by the end of stimulation. Day 5 median follicle size predicted median follicle size on subsequent ultrasound scans (*r*^2^ = 0.58–0.62; *p* < 0.0001), and hence time to oocyte maturation trigger (*r*^2^ = 0.22, *P* < 0.0001). Insufficient rFSH starting dose that required >5% dose-increase was associated with increased variability in follicle size on the day of oocyte maturation trigger, and negatively impacted the number of mature oocytes retrieved.

**Conclusion:** Weight-adjusted rFSH dose correlates with follicular growth during ovarian stimulation. Early recruitment of follicles using a sufficient dose of rFSH from the start of stimulation was associated with reduced variability in follicle size at time of oocyte maturation trigger and an increased number of mature oocytes retrieved.

## Introduction

IVF treatment is increasingly used to help subfertile couples to conceive, with the number of cycles carried out annually increasing by ~5% per year (1). However, 7.1% of cycles commenced in the UK did not result in retrieval of oocytes and no embryos were formed in 11.9% of cycles (2). A key factor in the success of assisted reproductive treatments is the induction of sufficient follicular growth by recombinant FSH (rFSH) administration, whilst concurrently avoiding excessive stimulation that could lead to an uncontrolled response.

In the physiological human menstrual cycle, a small (~30%) rise in serum FSH level, due to reduced estradiol negative feedback during the follicular phase (3), is sufficient to initiate growth of the most sensitive ovarian follicles (4, 5). As follicles grow, they produce estradiol and inhibin B, which negatively feedback on FSH secretion to restrict the “FSH window”—the period during which the FSH level remains above the threshold to prevent atresia of non-dominant follicles (4–7). In IVF cycles, a supraphysiological dose of rFSH is administered to maintain serum FSH levels above this threshold, to extend the FSH-window and promote multi-follicular growth (8, 9). An insufficient dose of rFSH negatively impacts on the number of follicles recruited and thus the number of oocytes retrieved. Conversely, an excessive dose of rFSH can lead to over-recruitment of follicles, increasing the risk of the potentially life-threatening complication ovarian hyperstimulation syndrome (OHSS), if hCG is used to induce oocyte maturation (10). Hence, appropriate rFSH dosing is regarded as a key treatment decision affecting both the success and safety of IVF treatment (11).

Selecting the optimal starting dose of rFSH is challenged by the interindividual variation in response to the same dose. Clinicians use clinical experience as well as baseline variables such as age, antral follicle count (AFC), serum anti-Mullerian hormone (AMH), body mass index (BMI), and follicular phase serum FSH level in selecting an appropriate rFSH dose in patients presenting for their first treatment cycle (12, 13). Due to the variability in response, a dominant factor in choosing rFSH dose in patients presenting for a second IVF cycle is the ovarian response in a previous cycle (12, 14). The starting dose of rFSH usually ranges between 100 and 450 IU, with 150 IU being recommended for the average-responder. Algorithms and nomograms to predict the number of oocytes retrieved have been developed to help clinicians choose the appropriate starting rFSH dose (11, 12, 14–17). The CONSORT algorithm takes into account age, BMI, follicular phase serum FSH and AFC to predict the number of oocytes retrieved in normo-ovulatory women aged < 35 years, treated with a GnRH agonist co-treated stimulation protocol (17). The PIVET algorithm recommends a rFSH starting dose based on age and AFC with adjustment for AMH, BMI, smoking and pre-treatment serum FSH level aiming to yield 8–12 oocytes in all women (11).

The majority of dosing algorithms relate rFSH dose to the number of oocytes retrieved, which assumes a homogenous response to the trigger of oocyte maturation (17). However, additional factors such as follicle size on the day of trigger, interval between trigger to oocyte retrieval and endocrine response to trigger also influence the number of oocytes retrieved, over and above response to rFSH dose (18). Antral follicle count (AFC) can be regarded as an estimate of the follicle pool that could respond to rFSH stimulation. Thus, researchers have evaluated the success of follicular growth in relation to AFC by calculating the “follicular output rate” (FORT) (i.e., number of follicles of 16–22 mm on the day of trigger expressed as a percentage of AFC) (19, 20). Others have reported the “follicle to oocyte index (FOI),” which is the number of oocytes expressed as a proportion of the antral follicle count (21).We have previously reported that follicles of 12–19 mm on the day of trigger are most likely to yield mature oocytes (22). Thus, in the current study we evaluated whether the starting rFSH dose predicted the proportion of antral follicles that resulted in follicles of 12–19 mm by the end of stimulation and investigated the relationship between rFSH dose and follicular growth during ovarian stimulation in 1,034 IVF cycles.

## Materials and Methods

We performed a single-center retrospective cohort study evaluating data-records from 1,034 GnRH antagonist co-treated IVF cycles. The study was conducted at the Wolfson Fertility Centre, Hammersmith Hospital, UK, between January 2012 and January 2016 (inclusive) in patients who had ovarian stimulation by Gonal F (Merck Serono, Geneva, Switzerland). Exclusion criteria were: use of additional/alternative medications for ovarian stimulation including urinary FSH, recombinant LH, or long-acting FSH preparations, or missing data on rFSH doses. Women were not excluded for having PCOS, endometriosis or for meeting Bologna/Poseidon criteria (23–25). A subset of cycles (*n* = 175) were completed during clinical trials investigating the use of kisspeptin as an oocyte maturation trigger and had more intensive monitoring of endocrine values such as serum FSH and LH during ovarian stimulation (26–28).

Baseline demographic data such as height, weight, ethnicity, pre-treatment serum FSH (iU/L), LH (iU/L), AMH (pmol/L), estradiol (pmol/L) and total AFC on transvaginal ultrasound were collated. For the vast majority of patients, follicular phase AFC and FSH measurements occurred within 3 months of commencing stimulation. AFC was measured manually using 2D ultrasound. Follicle sizes were assessed by transvaginal ultra-sonographic measurement (Toshiba Xario Prime, Crawley, UK) conducted by up to nine experienced IVF physicians/ultrasonographers at Hammersmith IVF unit over the 4-year study period. The unit protocol is to conduct an ultrasound scan after 4–5 days of rFSH. Starting doses of rFSH (between 100 IU and 450 IU daily) were selected based on age, AFC and physician experience. Patients may have had either fixed (day 5 of rFSH) or flexible start (lead follicle of 14 mm) of GnRH antagonist administration due to a change in unit policy during the time of the study. Two thirds of patients started GnRH antagonist on day 5 of rFSH, 10% on day 7–8, 11% on day 9, and 13% on days 10–12. We collated data on rFSH doses throughout ovarian stimulation, follicle counts and sizes at each monitoring ultrasound scan. All follicle diameters at each scan were individually recorded and a median follicle size was calculated. Follicle sizes <6 mm in diameter were recorded as measuring 5 mm during analysis. Preliminary linear regression analysis suggested that an age of 35 years represented an inflection point for response to rFSH. Evaluation of follicular response following rFSH was assessed both in a subset of patients with predicted ‘good response’ (age 18–34 years and AFC >15) and those with predicted “poorer response” (age ≥35 years and AFC ≤15). Remaining patients were classified as intermediate responders (either age 18–34 years and AFC ≤15 or age ≥35 years and AFC >15).

### Study Approval

Patients who received kisspeptin to trigger oocyte maturation from randomized clinical trials approved by the Hammersmith and Queen Charlotte's Research Ethics Committee, London, UK (26–28). The trials were registered on the National Institutes of Health Clinical Trials database (NCT01667406) and performed in accordance with the Declaration of Helsinki. All patients included in the study were treated at the IVF Unit at Hammersmith Hospital, London, UK, under a license from the UK Human Fertilization and Embryology Authority.

### IVF Stimulation Protocol

Recombinant FSH (rFSH) (100–450 IU Gonal F, Merck Serono, Geneva, Switzerland) was used to induce follicular growth. Gonal F was started from day 2 or 3 in GnRH antagonist co-treated ICSI cycles. Premature ovulation was prevented by a GnRH antagonist- either cetrorelix (0.25 mg, Cetrotide, Merck Serono, Middlesex, UK) or ganirelix (0.25 mg Orgalutran, Merck Sharp & Dohme Ltd, Hertfordshire, UK). The trigger was administered once 3 ovarian follicles were ≥18 mm in diameter and oocyte retrieval was conducted 36 h thereafter. The trigger of oocyte maturation was either hCG, choriogonadotrophin alpha (0.25 mg, Ovitrelle; Merck Serono, Feltham, UK), GnRH agonist—buserelin acetate (2 mg, Suprecur, Sanofi-Aventis, Guildford, UK), or kisspeptin-54 (6.4–12.8 nmol/kg as a single bolus, or 19.2 nmol/kg as a split-dose over 10 h, Bachem Holding AG, Bubendorf, Switzerland) administered subcutaneously (26–28). Data from only one IVF cycle per woman was used during the analysis.

### Assays Used

Serum LH, FSH, estradiol, and progesterone were measured using automated chemiluminescent immune immunoassays (Abbott Diagnostics, Maidenhead, UK). Interassay coefficients of variation were as follows: LH, 3.4%; FSH, 3.5%; estradiol, 3.4%; progesterone, 1.8%. Limits of detectability for each assay were as follows: LH 0.07 mIU/ml; FSH 0.05 mIU/ml; estradiol 70 pmol/l (19 pg/mL); progesterone 0.3 nmol/L (0.1 ng/mL). AMH was measured using an enzyme linked immunosorbent assay (Beckman Coulter Inc, Brea, CA, USA). The reference range was 2.2–48.5 pmol/L (0.3–6.8 ng/mL). The lower limit of detection was 0.6 pmol/l (0.08 ng/mL). The interassay coefficient of variation was 4.6%; the intraassay coefficient of variation was 4.0%.

### Statistical Analysis

Statistical analysis was performed using GraphPad PRISM version 7.0, STATA version 14.0 and R version 3.5.1. D'Agostino & Pearson normality test was used to assess data distribution. Normally distributed data is presented as mean ±standard deviation (SD), and non-parametric data as median with interquartile range (IQR). Non-parametric paired data for two groups was compared by the Wilcoxon signed rank test. Non-parametric unpaired data with two groups was compared using Mann-Whitney *U* test. For parametrically distributed data with multiple groups, one-way analysis of variance (ANOVA) was used, whereas for non-parametrically distributed data, the Kruskal-Wallis test was used. Simple linear regression modeling was performed with Pearson correlation coefficients for parametrically distributed data and Spearman correlation coefficients for non-parametrically distributed data. Categorical variables are presented as count (percentage; %). Multivariate generalized linear regression was used to assess the relationship between dose of Gonal F and follicular growth with the following variables assessed: age, AFC and pre-treatment serum FSH level. R^2^ coefficients of determination were derived using the formula R^2^ = 1 – (residual deviance)/(null deviance). A *p* < 0.05 was regarded as signifying statistically significance.

## Results

### Baseline Characteristics

Overall, 1034 GnRH antagonist co-treated cycles were included, including 354 predicted “good responders” (age 18–34 years and AFC >15) and 183 predicted “poor responders” (AFC ≤ 15 & age ≥35 years; Table 1). As serum FSH and LH are not routinely measured in IVF/ICSI cycles, this data was only available in a subset of patients triggered with kisspeptin (*n* = 175).

**Table 1:** Baseline characteristics.

		**All cycles**	**Potential to respond to rFSH**			***P*-value**
			**Good responders** **(AFC >15 & age 18–34 years)**	**Intermediate responders** **(either AFC≤15 or age ≥35 yrs)**	**Poorer responders** **(AFC≤15 & age ≥35 yrs)**	
*n*		1,034	354	497	183	-
Age (years)		35 [32–39]	32 [30–33]	38 [36–41]	40 [38–43]	*<0.0001*
Weight (kg)		63.9 [56.7–71.9]	63.9 [56.5–70.0]	63.5 [57.0–72.1]	63.0 [55.0–72.0]	0.96
BMI (kg/m^2^)		23.7 [21.5–27.0]	23.8 [21.8–27.1]	23.6 [21.3–27.3]	24.0 [21.0–26.0]	0.65
Starting rFSH dose (iU)		150.0	112.5	150.0	300.0	*<0.0001*
		[112.5–187.5]	[112.5–150]	[112.5–150]	[225–300]	
Starting rFSH dose per kg (iU/kg)		2.20 [1.80–2.8]	1.95 [1.66–2.30]	2.25 [1.85–2.78]	4.05 [3.13–5.00]	*<0.0001*
Total Antral Follicle Count (AFC)		25 [17–34]	30 [24–40]	26 [20–34]	10 [7–13]	*<0.0001*
Number of oocytes		12 [8–17]	12 [9–17]	12 [9–18]	7 [4–11]	*<0.0001*
Number of mature oocytes		9 [6–13]	10 [7–14]	10 [7–14.5]	6 [3–8]	*<0.0001*
Number of zygotes		7 [4–10]	8 [4–11]	7 [4–10]	4 [2–6]	*<0.0001*
Clinical Pregnancy Rate (%)		50.2%	54.5%	52.2%	39.5%	*0.006*
Live Birth Rate (%)		40.1%	45.9%	41.9%	28.2%	*0.001*
Median follicle size (mm)	Day 4	6.0 [6.0–7.0]	6.0 [5.3–6.0]	6.8 [6.0–7.5]	7.0 [5.5–7.5]	*0.0011*
	Day 5	7.0 [6.0–8.0]	7.0 [6.0–8.0]	7.0 [6–8.5]	8.3 [7.0–10.0]	*<0.0001*
	Day 7	9.0 [8.0–11.0]	9.0 [7.0–10.5]	9.0 [8.0–11.0]	10.3 [9.0–12.0]	*0.0003*
	Day 9	12.0 [10.0–13.0]	11.0 [10.0–13.0]	12.0 [10.0–13.0]	13.0 [11.0–14.0]	*<0.0001*
Follicle count on final scan		23.0 [16.0–32.0]	27.0 [21.0–36.0]	24.0 [18.0–33.0]	12.0 [8.0–17.0]	*<0.0001*
Day 5 serum estradiol (pmol/L)		1131 [672–1958]	918 [545–1677]	1276 [710–2064]	1099 [642–1767]	0.015
Ethnicity	Afro-Caribbean	62 (6.0%)	14 (4.1%)	31 (6.7%)	11 (6.4%)	-
	Caucasian	530 (51.0%)	190 (56.9%)	243 (52.0%)	97 (56.1%)	-
	Middle Eastern	14 (1.0%)	6 (1.8%)	8 (1.7%)	0	-
	East Asian	14 (1.0%)	1 (0.3%)	8 (1.7%)	5 (2.9%)	-
	South Asian	312 (31.0%)	107 (32.0%)	152 (32.6%)	54 (31.2%)	-
	Other	101 (10.0%)	16 (4.8%)	24 (5.2%)	6 (3.5%)	-

*Median [IQR] for continuous variables and number (%) for categorical variables of baseline characteristics are presented for all GnRH antagonist co-treated cycles (n = 1034). These are further subdivided into predicted “good responders” (antral follicle count (AFC) >15 and age 18–34 years; n = 354), “intermediate responders” (AFC ≤15 or age ≥35; n = 497) and “poorer responders” (AFC ≤15 and age ≥35 years; n = 183). Groups were compared by the Kruskal Wallis test. Categorical variables are presented as count (percentage; %) and compared by the Chi squared Test. Font in italics denotes significance*.

### Dose-Adjustments

Of the 1,034 GnRH antagonist co-treated cycles included, 270 (26%) had an average rFSH dose during treatment that was at least 5% higher compared to the starting dose and 82 (8%) had an average dose that was at least 5% lower than the starting dose, whereas the remaining 682 (66%) received an average dose within ±5% of the starting dose.

### Serum FSH and Follicular Growth

**Figure 1:**
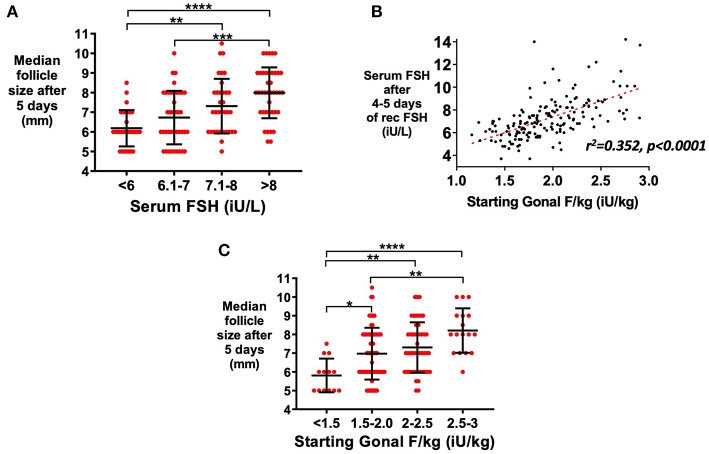
Relationship between serum FSH level and follicle growth. Serum AMH and FSH levels were available in a subset of patients triggered with kisspeptin (serum AMH >10 pmol/L; *n* = 147). **(A)** Mean (±SD) of median follicle size on ultrasound by categories of steady state serum FSH levels (iU/L) at 5 days after starting recombinant FSH dose (rFSH) is presented (*n* = 144; **A**). Categories were compared by one-way ANOVA with *post hoc* Tukey's multiple comparisons test. **(B)** Dose of rFSH adjusted for body weight (iU/kg) predicted serum FSH level (*n* = 166) by simple linear regression: Day 4–5 serum FSH level = 2.79 x starting rFSH dose (iU/kg) + 1.8, *r*^2^ = 0.352, *p* < 0.0001. **(C)** Mean (±SD) of median follicle size on ultrasound by categories of starting rFSH adjusted for weight (iU/kg) at 5 days after starting rFSH is presented (*n* = 147). Categories were compared by one-way ANOVA with *post hoc* Tukey's multiple comparisons test. *p < 0.05, **p < 0.01, ***p < 0.001, ****p < 0.0001.

Serum FSH level at steady-state measured after 5 days of rFSH administration was associated with median follicle size on ultrasound (Figure 1A). Serum FSH levels were predicted by rFSH dose-adjusted for body weight (*r*^2^ = 0.352, *P* < 0.001; Figure 1B). Recombinant FSH dose-adjusted for body weight (IU per kg) thus also predicted median follicle size following 5 days of treatment (Figure 1C). To achieve the same median follicle size after 5 days of stimulation, a higher dose of rec rFSH was needed if AMH levels were <40 pmol/L in comparison to those with higher AMH levels (≥40 pmol/L; Supplemental Figure 1A). Stratifying patients by screening serum FSH level did not improve prediction of serum FSH level, suggesting that measured serum FSH level predominantly represents exogenous rFSH dosing (Supplemental Figure 1B). Serum FSH levels were not altered by GnRH antagonist administration (*p* = 0.937; Supplemental Figure 1C), whereas serum LH levels decrease by ~50% (Supplemental Figure 1D; *p* < 0.0001).

### Recombinant FSH and Follicular Growth

**Figure 2:**
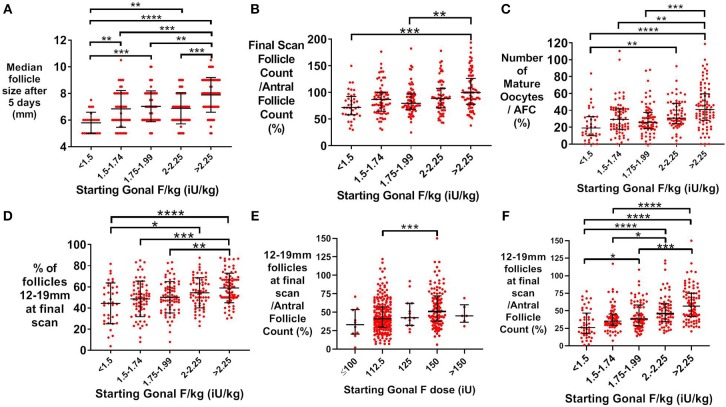
Effect of starting rFSH dose on follicle growth in predicted “good responders” (AFC>15 and age 18–34 years; *n* = 354). **(A)** Mean (±SD) starting Gonal F/kg is positively associated with median follicle size on ultrasound (mm) after 5 days of rFSH treatment. Categories were compared by one-way ANOVA with *post hoc* Tukey's multiple comparisons test (*n* = 241). **(B)** Median (±IQR) starting dose of Gonal F/kg is positively associated with the proportion of antral follicles recruited by the final ultrasound scan during controlled ovarian stimulation. Categories were compared by the Kruskal-Wallis test with *post hoc* Dunn's multiple comparisons test (*n* = 324). **(C)** Median (±IQR) starting dose of rFSH adjusted for weight (iU/kg) is associated with the proportion of antral follicles that result in a mature oocyte being retrieved. Categories were compared by the Kruskal-Wallis test with *post hoc* Dunn's multiple comparisons test (*n* = 324). **(D)** Mean (±SD) of the proportion of 12–19 mm follicles at the final scan is positively associated with categories of starting rFSH dose adjusted for body weight. Categories were compared by one-way ANOVA with *post hoc* Tukey's multiple comparisons test (*n* = 324). **(E)** Median (±IQR) of the proportion of 12–19 mm follicles at the final scan as a function of the antral follicle count is positively associated with categories of starting rFSH dose adjusted for body weight. Categories were compared by the Kruskal-Wallis test with *post hoc* Dunn's multiple comparisons test (*n* = 324). **(F)** Median (±IQR) of the proportion of 12–19 mm follicles at the final scan as a function of the antral follicle count is positively associated with categories of unadjusted starting rFSH dose. Categories were compared by the Kruskal-Wallis test with *post hoc* Dunn's multiple comparisons test (*n* = 324). *p < 0.05, **p < 0.01, ***p < 0.001, ****p < 0.0001.

In patients predicted to be “good responders” (aged 18–34 years and AFC >15), median follicle size following 5 days of treatment was associated with weight-adjusted rFSH dose (Figure 2A). The proportion of antral follicles recruited by the final ultrasound scan during stimulation was also increased with dose of rFSH (Figure 2B), as was the number of mature oocytes expressed as a proportion of antral follicle count (Figure 2C). The proportion of follicles that were within the range for follicle size most likely to yield a mature oocyte on day of trigger (12–19 mm in diameter) significantly increased with starting dose of rFSH (Figure 2D). Furthermore, the number of follicles of 12–19 mm in diameter on the day of trigger expressed as a proportion of the AFC increased with weight-adjusted dose of FSH (Figure 2E), with greater discrimination than with absolute rFSH doses (Figure 2F).

Median follicle size after 5 days of rFSH was a useful measure of ovarian response, positively predicting median follicle size on day 7 (*r*^2^ = 0.58, *p* < 0.0001; Supplemental Figure 2A), then in turn on day 9 (*r*^2^ = 0.62, *p* < 0.0001; Supplemental Figure 2B), and thus time to oocyte maturation trigger (*r*^2^ = 0.22, *p* < 0.0001; Supplemental Figure 2C). The dose-response relationship was also apparent in patients considered intermediate responders (either AFC ≤ 15 or age ≥35 years) (Supplemental Figure 3), but was lost in patients were predicted to be “poorer responders” receiving doses more than 2.25 iU/kg (Supplemental Figure 4).

Multivariate linear regression models were used to investigate the relationship between rFSH dose and median follicle size (Table 2) and the proportion of antral follicles recruited (Table 3), adjusted for relevant baseline variable factors. Starting rFSH dose (IU/kg) significantly predicted day 5 median follicle size when adjusted for age, total AFC and pre-treatment serum FSH level (*r*^2^ = 0.152, *p* < 0.00001, *n* = 630; Table 2). Starting rFSH dose (IU/kg) also predicted the proportion of antral follicles recruited by the final scan when adjusted for age, total AFC and pre-treatment serum FSH level (*r*^2^ = 0.147, *p* < 0.00001, *n* = 1,027; Table 3).

**Table 2:** Median follicle size after 5 days.

**Variable**	**Coefficient (β)**	***p*-value**
Starting gonal F/kg	0.29	0.001
Antral follicle count	−0.02	0.001
Pre-treatment screening FSH	−0.001	0.75
Age	0.023	0.11
Constant	7.27	0.0001

*Multivariate linear regression analysis of median follicle size on day 5 of rFSH treatment and starting Gonal F dose per kg, adjusted for antral follicle count (AFC), age, and pre-treatment serum FSH level (n = 630); r^*2*^ = 0.152, p < 0.0001*.

**Table 3:** Proportion of antral follicles recruited.

**Variable**	**Coefficient (β)**	***p*-value**
Starting gonal F/kg	0.09	0.0001
Antral follicle count	−0.01	0.0001
Pre-treatment screening FSH	−0.001	0.94
Age	−0.01	0.23
Constant	1.4946	0.0001

*Multivariate linear regression analysis of proportion of antral follicles recruited by final ultrasound scan before administration of oocyte maturation trigger and starting rFSH dose per kg, adjusted for antral follicle count (AFC), age, and pre-treatment serum FSH level (n = 774); r^*2*^ = 0.147, p < 0.0001*.

### Starting Dose of rFSH and Variability of Follicle Size by Day of Trigger

**Figure 3:**
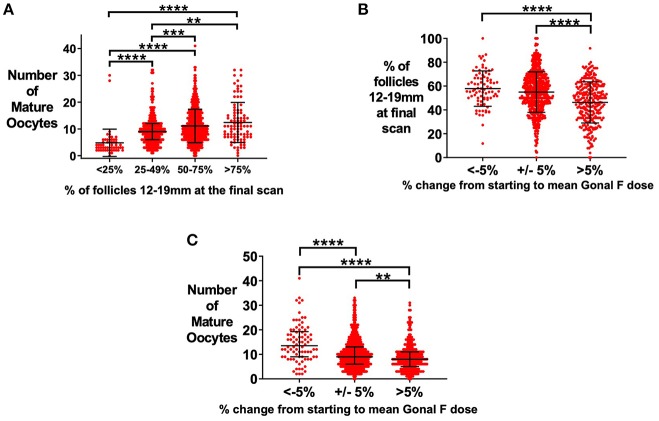
Effect of starting rFSH dose on variability in follicle size on day of trigger. **(A)** Median (±IQR) of the number of mature oocytes is presented by proportion of follicles sized 12–19 mm at the final ultrasound scan prior to administration of oocyte maturation trigger. Categories were compared by the Kruskal-Wallis test with *post hoc* Dunn's multiple comparisons test (*n* = 1029). **(B)** Increasing an insufficient starting rFSH dose during the cycle resulted in a reduced proportion of follicles sized 12–19 mm (*n* = 1034). Mean (±SD) is presented. Categories were compared by one-way ANOVA with *post hoc* Tukey's multiple comparisons test. **(C)** Increasing an insufficient starting rFSH dose during the cycle resulted in fewer mature oocytes retrieved. Median (±IQR) number of mature oocytes is presented. Categories were compared by the Kruskal-Wallis test with *post hoc* Dunn's multiple comparisons test (*n* = 1034). **p < 0.01, ***p < 0.001, ****p < 0.0001.

An increased proportion of follicles of 12–19 mm on the day of oocyte maturation trigger was associated with more mature oocytes retrieved (Figure 3A) (22). Median follicle size of 14–16 mm on day of oocyte maturation trigger was associated with the highest proportion of follicles being sized between 12 and 19mm (Supplemental Figure 1E). The dose of rFSH was altered in some patients during the cycle if follicular growth was not as anticipated. Patients who required an increase in the average dose of rFSH by more than 5% compared to the starting dose, had increased variability of follicle size with a lower proportion of follicles within 12–19 mm on the final ultrasound scan before trigger (Figure 3B), and fewer mature oocytes retrieved (Figure 3C).

### Serum Estradiol as a Marker of Follicular Response

**Figure 4:**
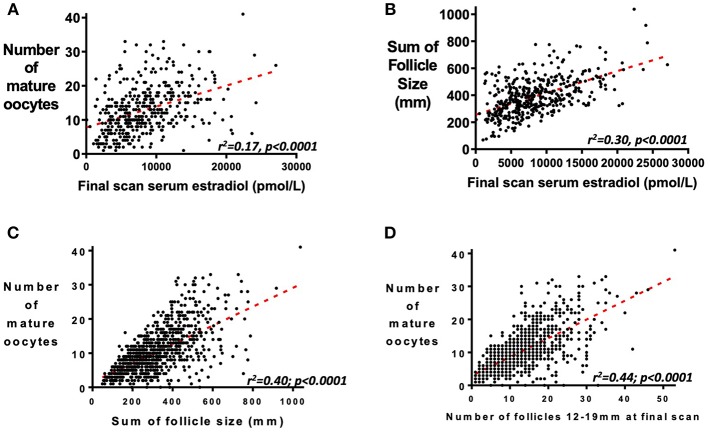
Relationship between serum estradiol, follicle size and number of mature oocytes retrieved. **(A)** Serum estradiol (pmol/L) on the day of the final scan before administration of the trigger of oocyte maturation predicts the number of mature oocytes subsequently retrieved (*n* = 419; *r*^2^ = 0.17, *P* <0.0001). **(B)** Serum estradiol (pmol/L) at the final scan before administration of the trigger of oocyte maturation also correlates with the cumulative sum of follicle sizes (mm) of all follicles by ultrasound scan (*n* = 419; *r*^2^ = 0.30, *P* < 0.0001). Whilst the sum of follicle sizes on the final scan before administration of oocyte maturation trigger predicted the number of mature oocytes retrieved (**C**, *n* = 1034; *r*^2^ =0.40, *P* < 0.0001), this was better predicted by the number of follicles sized 12–19 mm in diameter (**D**, *n* = 1031; *r*^2^ = 0.44, *P* < 0.0001).

Serum estradiol measurement on the day of final scan is predictive of the number of mature oocytes retrieved (Figure 4A, *r*^2^ = 0.17, *p* < 0.0001) and the cumulative sum of all follicle sizes on the final scan before oocyte maturation trigger (Figure 4B, *r*^2^ = 0.30, *p* < 0.0001). The cumulative sum of follicle sizes is a better predictor of the number of mature oocytes retrieved than serum estradiol on the day of the final scan (Figure 4C, *r*^2^ = 0.40, *p* < 0.0001). However, as previously reported, the number of follicles sized between 12 and 19 mm is a better predictor still of the number of mature oocytes retrieved (Figure 4D, *r*^2^ = 0.44, *p* < 0.0001) (22).

Serum LH was positively associated with serum estradiol both after 5 days of rFSH treatment (Supplemental Figure 5A) and on the final morning before oocyte maturation trigger administration (Supplemental Figure 5B). A higher serum LH was associated with a greater BMI both before commencement of the GnRH antagonist on day 5 of rFSH treatment (*p* < 0.0001) (Supplemental Figure 5C) and 2 days thereafter (*p* = 0.0015; Supplemental Figure 5D).

## Discussion

We investigated the relationship between rFSH dose and follicle growth in data from 1,034 IVF cycles. Preliminary analysis revealed that recombinant FSH dose adjusted for body weight (IU per kg) was associated with steady-state serum FSH levels and median follicle size following 5 days of rFSH treatment. Median follicle size after 5 days of rFSH was a useful measure, positively predicting median follicle size on subsequent scans and thus time to administration of oocyte maturation trigger. Once antral follicles are recruited to grow, they become less FSH-dependent for their continued growth and accrue LH receptors (29). Indeed, this is also reflected by the isoform of FSH changing during the follicular phase to become more acidic and less bioactive (30).

Antral follicles represent a pool of follicles capable of being recruited to grow if sufficient FSH exposure is provided. The differential sensitivity of individual antral follicles within a cohort, enables the success of step-up protocols for inducing mono-follicular growth in ovulation induction cycles, by gradually achieving the minimum threshold for growth of the most sensitive follicles (31). In the current study, the starting rFSH dose predicted the proportion of antral follicles that were recruited by the final scan before administration of the trigger of oocyte maturation. In some women, more follicles were recruited by the end of stimulation than pre-stimulation antral follicles. Possible explanations for this observation could include that secondary waves of antral follicles were recruited later during the stimulation phase, or alternatively that measurement error in the quantification of antral follicle count could have underestimated the true antral follicle count.

During a multi-variate analysis, variables identified as being relevant were consistent with those identified to influence oocyte number in the CONSORT algorithm (BMI, AFC, age) (32, 33). Accordingly, these data enable reproductive endocrinologists to individualize follicle recruitment based on treatment aims for an individual patient. For example, follicle recruitment in a woman at high risk of OHSS can be decreased if hCG is to be used as the trigger of oocyte maturation, as per the intention of the PIVET protocol (11). Oocyte number expressed as a proportion of antral follicle count was associated with starting rFSH dose, in keeping with data from other FSH preparations (34). Alternatively, these data can inform rFSH dosing to maximize follicular recruitment, if an alternate trigger to hCG is used to induce oocyte maturation in order to mitigate the risk of OHSS (35).

In accordance with the published literature, we observed that the dose-response relationship was lost in anticipated poor responders (AFC ≤ 15 & age ≥ 35 years) receiving doses of rFSH over 2.25iU/kg (36, 37). Thus, 1.5 iU/kg can be regarded as a low dose of rFSH and 2.25 iU/kg as an upper threshold dose, beyond which minimal further follicular response is anticipated, in keeping with similar data using follitropin delta (38).

Serum estradiol is used as a marker of adequate ovarian response by some clinicians, and rFSH dose increased if serum estradiol is deemed suboptimal, even if follicle growth appears adequate. However, this relationship appears to be indirect; serum estradiol at the end of stimulation was correlated with the cumulative sum of follicle sizes on the day of trigger, as well as the number of mature oocytes subsequently retrieved. Nevertheless, the number of follicles sized 12–19 mm at the end of stimulation was a better predictor of the number of mature oocytes retrieved than serum estradiol level (Figure 4) (22).

We investigated the impact of dose-adjustment during stimulation. We hypothesized that an insufficient dose of rFSH could lead to recruitment of the most sensitive follicles, whilst the majority of follicles would remain dormant. A subsequent dose-increase would recruit another wave of follicles that would therefore result in increased variability of follicle size by the end of stimulation. We observed that a lower starting rFSH dose reduced the proportion of follicles sized 12–19 mm at the end of stimulation in keeping with data using a novel human rFSH (39) and with our previous data (22). Furthermore, the need to increase the mean dose of rFSH by more than 5% of the starting dose reduced the proportion of follicles of 12–19 mm at the end of stimulation, and this was associated with 5 fewer mature oocytes.

The rFSH preparation studied (Gonal F) has a terminal half-life of 1 day and reaches steady state levels after 3–4 days (40). Thus, in some units, a higher dose of rFSH is used for the first few days of stimulation to rapidly achieve adequate FSH levels and recruit all available follicles, before the dose is reduced to maintenance levels (step-down protocol). Our data is consistent with such an approach being logical in aiming to recruit follicles as a closely clustered cohort at the beginning of stimulation, thus reducing variability in follicle size and optimizing the action of the oocyte maturation trigger (22).

It has been established that there is insufficient evidence to suggest that rFSH dose significantly impacts live birth rates (37). This is logical, since providing that at least one good quality embryo is formed, there is no biological rationale for a difference in live birth rate due to rFSH dose (37, 41, 42). However, a higher rFSH dose was associated with a reduced chance of cycle cancellation due to insufficient ovarian response, albeit at the expense of an increased risk of OHSS (37, 41, 42). Importantly in predicted high response women, there remained significant proportions of women experiencing either poor or hyper-response at both doses examined (100IU rFSH: poor-response 34%, hyper-response 12%; 150IU of rFSH: poor-response 8%, hyper-response 38%) (41).

Strengths of our study are the inclusion of a large number of cycles examined at a single center, thereby limiting heterogeneity due to protocol differences amongst different reproductive endocrinology treatment centers. Furthermore, monitoring was conducted using the same ultrasound machines (Toshiba Xario Prime, Crawley, UK) and no more than 9 experienced IVF practitioners measured follicle size during the period of the study. Only data from cycles that used rFSH (Gonal F) alone were included, thus limiting variability due to other FSH preparations. Limitations of the study include its retrospective design, and that due to heterogeneity in day of monitoring ultrasound scans, not all cycles could be used in every analysis. Serum FSH, LH, and AMH levels were only measured for a subset of cycles and thus any benefit to using serum FSH level over rFSH dose adjusted for weight could not be ascertained. Serum AMH and AFC both have similar diagnostic accuracy as ovarian response markers (43), although AMH may be considered as more reproducible than AFC between centers (44). The majority of analyses assessed parameters of follicle growth and were thus unaffected by the trigger used, as these outcomes preceded administration of the trigger. For data which included the number of mature oocytes retrieved, sensitivity analyses confirmed that results were comparable regardless of trigger used. Prospective studies prescribing rFSH using a weight-based regimen and evaluating follicular growth are indicated to further overcome the limitations of the retrospective design. The use of 3-D ultrasound to track follicular development in future prospective studies could more accurately relate rFSH dose to follicular growth.

To conclude, we have identified a dose-response for starting rFSH dose to median follicle size following 5 days of treatment, and to the proportion of antral follicles recruited. Our data suggests that commencing stimulation with a sufficient rFSH dose from the start of the cycle reduces variability in follicle size to thus maximize the number of mature oocytes retrieved. Consistent with published data suggesting an upper threshold to useful FSH dosing, we find minimal further increase in response beyond a rFSH dose of 2.25iU/kg (37). These data reveal important physiological information on FSH requirements for follicular growth. In addition, these findings inform clinicians in selecting a suitable starting rFSH dose for their patients that is tailored to the treatment aims for that individual. However, future research is required to further evaluate the dose-response relationships identified in this study.

## Data Availability

The datasets generated for this study are available on request to the corresponding author.

## Ethics Statement

Patients who received kisspeptin to trigger oocyte maturation from randomized clinical trials approved by the Hammersmith and Queen Charlotte's Research Ethics Committee, London, UK (26–28). The trials were registered on the National Institutes of Health Clinical Trials database (NCT01667406) and all subjects gave written informed consent in accordance with the Declaration of Helsinki and Good Clinical Practice. All patients included in the study were treated at the IVF Unit at Hammersmith Hospital, London, UK, under a license from the UK Human Fertilization and Embryology Authority.

## Author Contributions

All authors provided contributions to study conception and design, acquisition of data or analysis and interpretation of data, drafting the article or revising it critically for important intellectual content, and final approval of the version to be published. Here are the most important contributions of each author: AA, RS, GT, AC, SL, RR, and WD designed the study. Data was collected by AP, AA, TH, GC, SC, PE, and MP. Analysis was carried out by AA, AP, and TK. WD takes final responsibility for this article.

### Conflict of Interest Statement

The authors declare that the research was conducted in the absence of any commercial or financial relationships that could be construed as a potential conflict of interest.

## Supplementary Material

Supplemental Figure 1 **(A)** Median starting dose of rFSH adjusted for weight (iU/kg) is presented by categories of median follicle size after 5 days of rFSH, stratified by pre-treatment serum AMH level (either AMH <40 pmol/L or AMH ≥40pmol/L) (*n* = 147). **(B)** Simple linear regression of serum FSH level (iU/L) on day 4–5 of rFSH treatment by starting rFSH dose per kg (iU/kg) stratified by pre-treatment follicular phase serum FSH level (iU/L); (*n* = 166). There is no difference in relationship between serum FSH level at 4–5 days after starting rFSH treatment and starting dose of rFSH per kg when stratifying by pre-treatment serum FSH level. **(C,D)** Serum FSH **(B)** (iU/L), and serum LH (iU/L) **(C)** are presented, before starting GnRH antagonist treatment (day 5 of cycle), and 2 days after starting GnRH antagonist treatment (day 7); (*n* = 166) (2 outlying data points not shown for serum LH). Paired values of serum FSH and of serum LH were compared by the Wilcoxon signed rank test. GnRH antagonist administration caused no significant change in serum FSH levels (*p* = 0.937) **(B)**, but a significant reduction in the serum LH level (*p* < 0.0001) **(C)**. **(E)** Median follicle size (median ±IQR) on the day of oocyte maturation trigger is associated with the proportion of 12–19 mm follicles at the final scan (*n* = 175). Categories were compared by the Kruskal-Wallis test with *post hoc* Dunn's multiple comparisons test. ^****^*p* < 0.0001.

Supplemental Figure 2 Median follicle size after 5 days of rFSH treatment predicts median follicle size on subsequent scans **(A,B)** and thus the day of oocyte maturation trigger **(C)** by simple linear regression. **(A)** Median follicle size (mm) on day 5 and on day 7 of rFSH treatment (*n* = 351): Day 7 median follicle size = 1.066^*^Day 5 median follicle size + 1.45, *r*^2^ = 0.58, *p* < 0.0001. **(B)** Median follicle size (mm) on day 7 and day 9 of rFSH treatment (*n* = 260): Day 9 median follicle size = 0.997^*^Day 7 median follicle size + 2.01, *r*^2^ = 0.62, *p* < 0.0001. **(C)** Day of oocyte maturation trigger by median follicle size (mm) on day 5 of rFSH treatment; (*n* = 632). Day of oocyte maturation trigger = −0.67^*^Day 5 median follicle size + 15.94, *r*^2^ = 0.22; *p* < 0.0001.

Supplemental Figure 3 Follicle growth in a subset of predicted “intermediate responders” (either AFC > 15 or age 18–35 years). Median follicle size after 5 days of rFSH treatment **(A)**, the proportion of the antral follicle count (AFC) recruited to grow **(B)**, and the proportion of the antral follicles that yield mature oocytes **(C)** increase with starting dose of rFSH (IU per kg). **(A)** There is an association between starting rFSH dose (iU/kg) and median follicle size after 5 days of rFSH treatment. Mean (±SD) is presented and categories were compared by one-way ANOVA with *post hoc* Tukey's multiple comparisons test (*n* = 193). **(B)** There is an association between starting rFSH dose (iU/kg) and the proportion of antral follicles recruited to grow by the final scan (*n* = 323). Median (±IQR) is presented and categories were compared by the Kruskal-Wallis test with *post hoc* Dunn's multiple comparisons test (1 outlier was not presented). **(C)** There is an association between starting rFSH dose (iU/kg) and the proportion of 12–19 mm follicles at the final scan (*n* = 327). Mean (±SD) is presented and categories were compared by one-way ANOVA with Tukey's multiple comparisons test. **(D)** There is an association between starting rFSH dose (iU/kg) and the proportion of antral follicles that yield a mature oocyte (*n* = 323). Median (±IQR) is presented. Categories were compared by the Kruskal-Wallis test with Dunn's multiple comparisons test. ^*^*p* < 0.05, ^**^*p* < 0.01, ^***^*p* < 0.001, ^****^*p* < 0.0001.

Supplemental Figure 4 Follicle growth in response to rFSH in a subset of predicted poorer responders (AFC≤15 and age≥35 years). **(A)** No significant differences were found between categories of starting rFSH dose (iU/kg) and median follicle size after 5 days (*n* = 45). Mean (±SD) is presented and categories were compared by one-way ANOVA with *post hoc* Tukey's multiple comparisons test. **(B)** No significant differences were observed between starting rFSH dose (iU/kg) and the proportion of the antral follicle count (AFC) recruited to grow (*n* = 120). Median (±IQR) is presented and categories were compared by the Kruskal-Wallis test with *post hoc* Dunn's multiple comparisons test. **(C)** No significant differences were observed between starting rFSH dose (iU/kg) and the proportion of 12–19 mm follicles at the end of stimulation (*n* = 120). Mean (±SD) is presented and categories were compared by one-way ANOVA with *post hoc* Tukey's multiple comparisons test. **(D)** No significant differences were observed between starting rFSH dose (iU/kg) and the proportion of antral follicles that yield a mature oocyte (*n* = 120). Median (±IQR) is presented and categories were compared by the Kruskal-Wallis test with *post hoc* Dunn's multiple comparisons test.

Supplemental Figure 5 **(A,B)** Both serum LH after 5 days of rFSH treatment (**A**; *n* = 166) and serum LH on the final morning before oocyte maturation trigger administration (**B**; *n* = 164) are positively associated with serum estradiol after 5 days of rFSH treatment. Median (±IQR) is presented and categories were compared by the Kruskal-Wallis test with *post hoc* Dunn's multiple comparisons test (*n* = 166). **(C,D)** A lower body mass index (BMI) is associated with lower serum LH levels both before (**C**; *n* = 167) and after (**D**; *n* = 131) administration of GnRH antagonist. ^*^*p* < 0.05, ^**^*p* < 0.01, ^****^*p* < 0.0001. Median (±IQR) is presented and categories were compared by the Mann Whitney *U* test (*n* = 167).
