# Supplementary figures and images for: FSH Requirements for Follicle Growth During Controlled Ovarian Stimulation
Source: Front Endocrinol (Lausanne). 2019 Aug 27;10:579. doi: 10.3389/fendo.2019.00579 (PMC6718557; doi:10.3389/fendo.2019.00579)

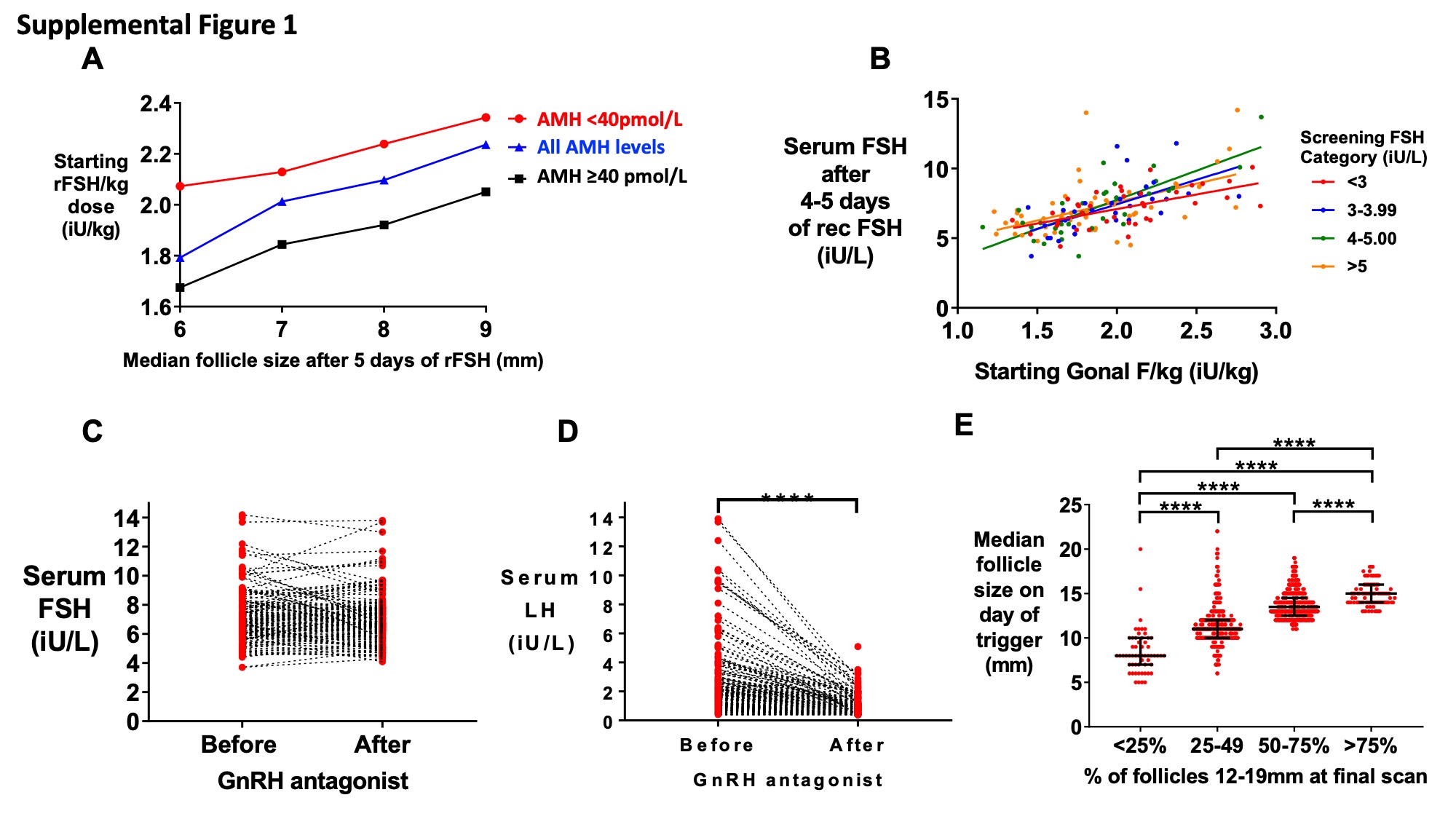

Supplement: Supplemental Figure 1 — (A) Median starting dose of rFSH adjusted for weight (iU/kg) is presented by categories of median follicle size after 5 days of rFSH, stratified by pre-treatment serum AMH level (either AMH <40 pmol/L or AMH ≥40pmol/L) (n = 147). (B) Simple linear regression of serum FSH level (iU/L) on day 4–5 of rFSH treatment by starting rFSH dose per kg (iU/kg) stratified by pre-treatment follicular phase serum FSH level (iU/L); (n = 166). There is no difference in relationship between serum FSH level at 4–5 days after starting rFSH treatment and starting dose of rFSH per kg when stratifying by pre-treatment serum FSH level. (C,D) Serum FSH (B) (iU/L), and serum LH (iU/L) (C) are presented, before starting GnRH antagonist treatment (day 5 of cycle), and 2 days after starting GnRH antagonist treatment (day 7); (n = 166) (2 outlying data points not shown for serum LH). Paired values of serum FSH and of serum LH were compared by the Wilcoxon signed rank test. GnRH antagonist administration caused no significant change in serum FSH levels (p = 0.937) (B), but a significant reduction in the serum LH level (p < 0.0001) (C). (E) Median follicle size (median ±IQR) on the day of oocyte maturation trigger is associated with the proportion of 12–19 mm follicles at the final scan (n = 175). Categories were compared by the Kruskal-Wallis test with post hoc Dunn's multiple comparisons test. ****p < 0.0001. [file Image_1.JPEG]

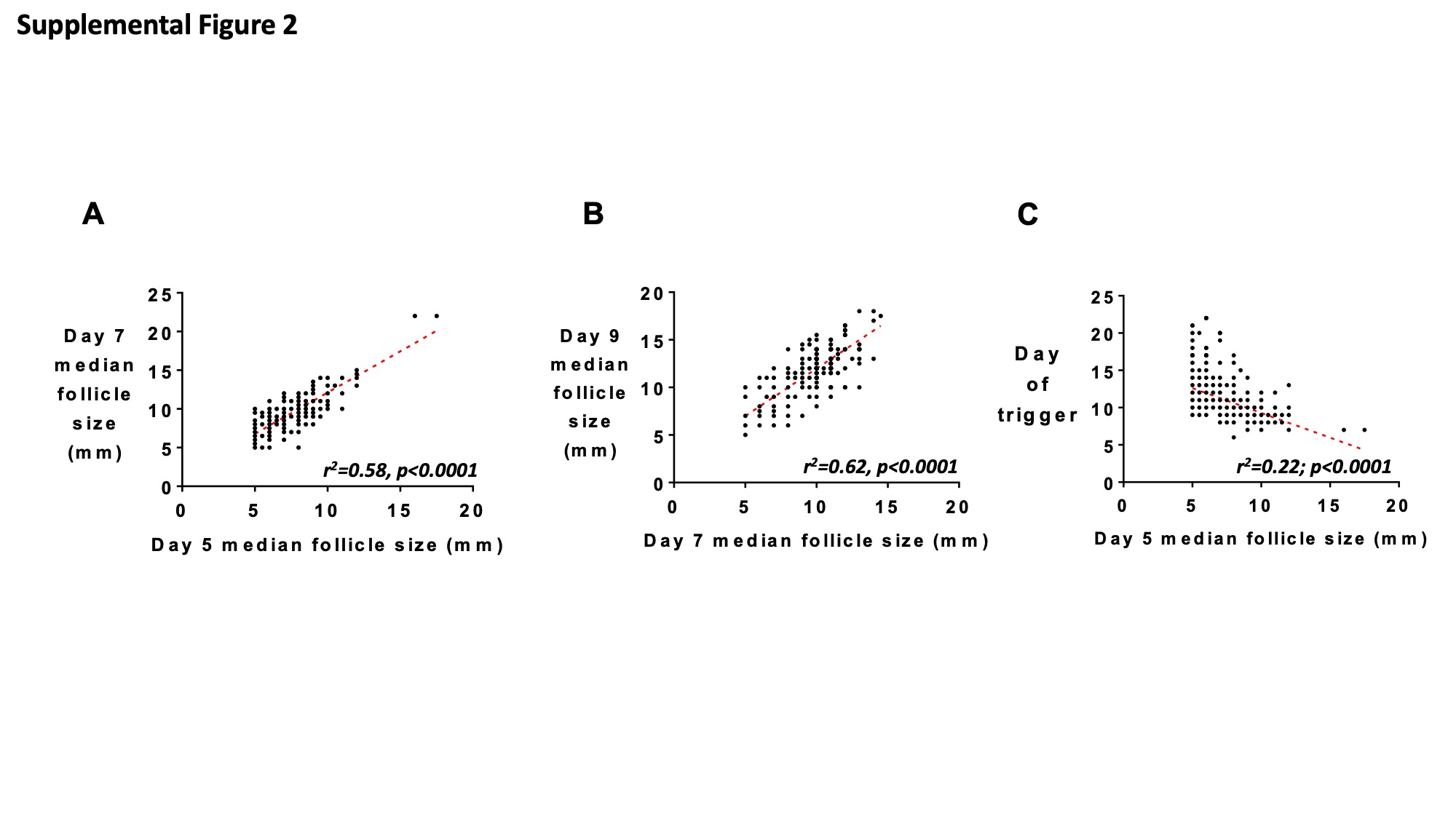

Supplement: Supplemental Figure 2 — Median follicle size after 5 days of rFSH treatment predicts median follicle size on subsequent scans (A,B) and thus the day of oocyte maturation trigger (C) by simple linear regression. (A) Median follicle size (mm) on day 5 and on day 7 of rFSH treatment (n = 351): Day 7 median follicle size = 1.066*Day 5 median follicle size + 1.45, r2 = 0.58, p < 0.0001. (B) Median follicle size (mm) on day 7 and day 9 of rFSH treatment (n = 260): Day 9 median follicle size = 0.997*Day 7 median follicle size + 2.01, r2 = 0.62, p < 0.0001. (C) Day of oocyte maturation trigger by median follicle size (mm) on day 5 of rFSH treatment; (n = 632). Day of oocyte maturation trigger = −0.67*Day 5 median follicle size + 15.94, r2 = 0.22; p < 0.0001. [file Image_2.JPEG]

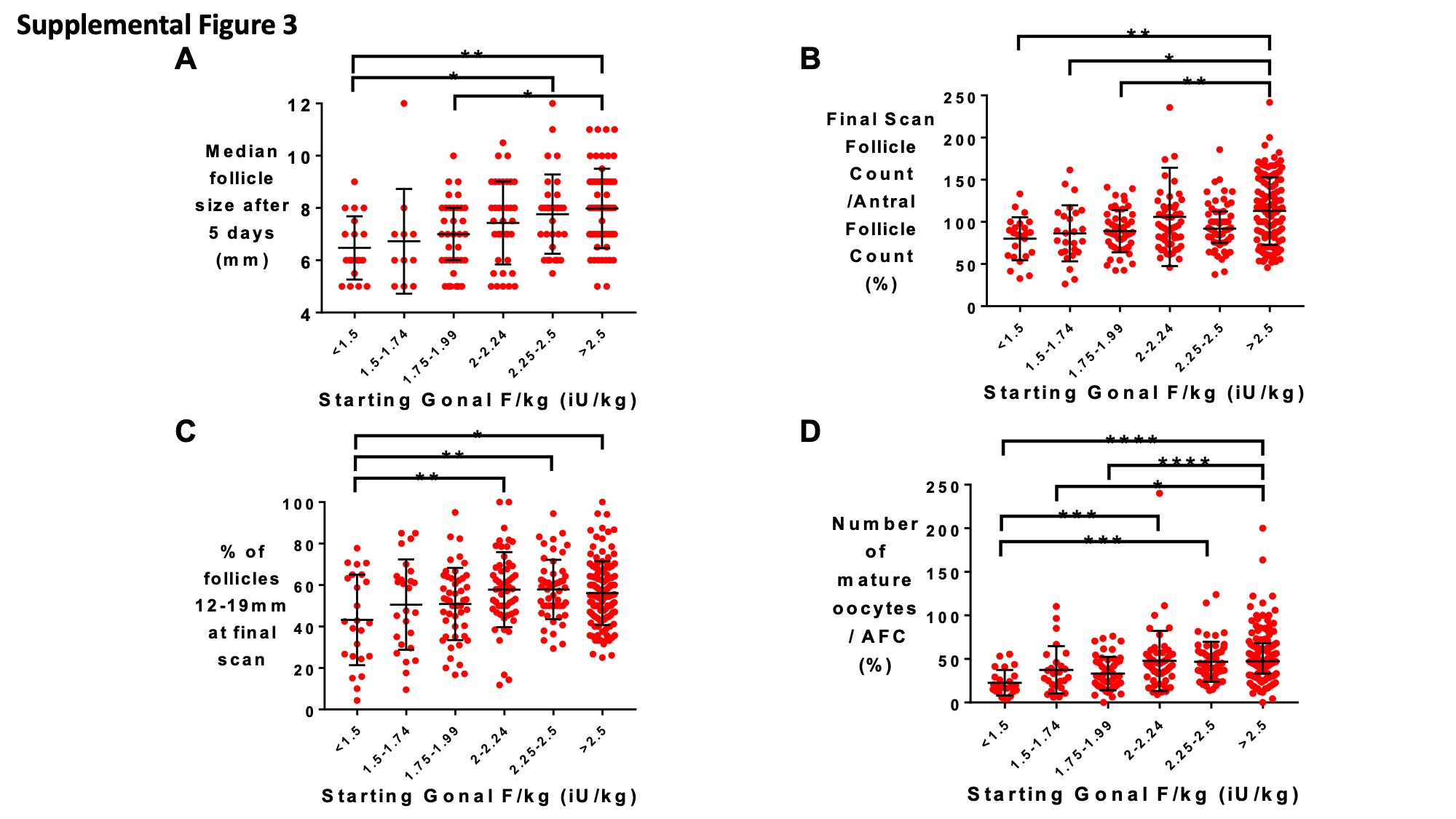

Supplement: Supplemental Figure 3 — Follicle growth in a subset of predicted “intermediate responders” (either AFC > 15 or age 18–35 years). Median follicle size after 5 days of rFSH treatment (A), the proportion of the antral follicle count (AFC) recruited to grow (B), and the proportion of the antral follicles that yield mature oocytes (C) increase with starting dose of rFSH (IU per kg). (A) There is an association between starting rFSH dose (iU/kg) and median follicle size after 5 days of rFSH treatment. Mean (±SD) is presented and categories were compared by one-way ANOVA with post hoc Tukey's multiple comparisons test (n = 193). (B) There is an association between starting rFSH dose (iU/kg) and the proportion of antral follicles recruited to grow by the final scan (n = 323). Median (±IQR) is presented and categories were compared by the Kruskal-Wallis test with post hoc Dunn's multiple comparisons test (1 outlier was not presented). (C) There is an association between starting rFSH dose (iU/kg) and the proportion of 12–19 mm follicles at the final scan (n = 327). Mean (±SD) is presented and categories were compared by one-way ANOVA with Tukey's multiple comparisons test. (D) There is an association between starting rFSH dose (iU/kg) and the proportion of antral follicles that yield a mature oocyte (n = 323). Median (±IQR) is presented. Categories were compared by the Kruskal-Wallis test with Dunn's multiple comparisons test. *p < 0.05, **p < 0.01, ***p < 0.001, ****p < 0.0001. [file Image_3.JPEG]

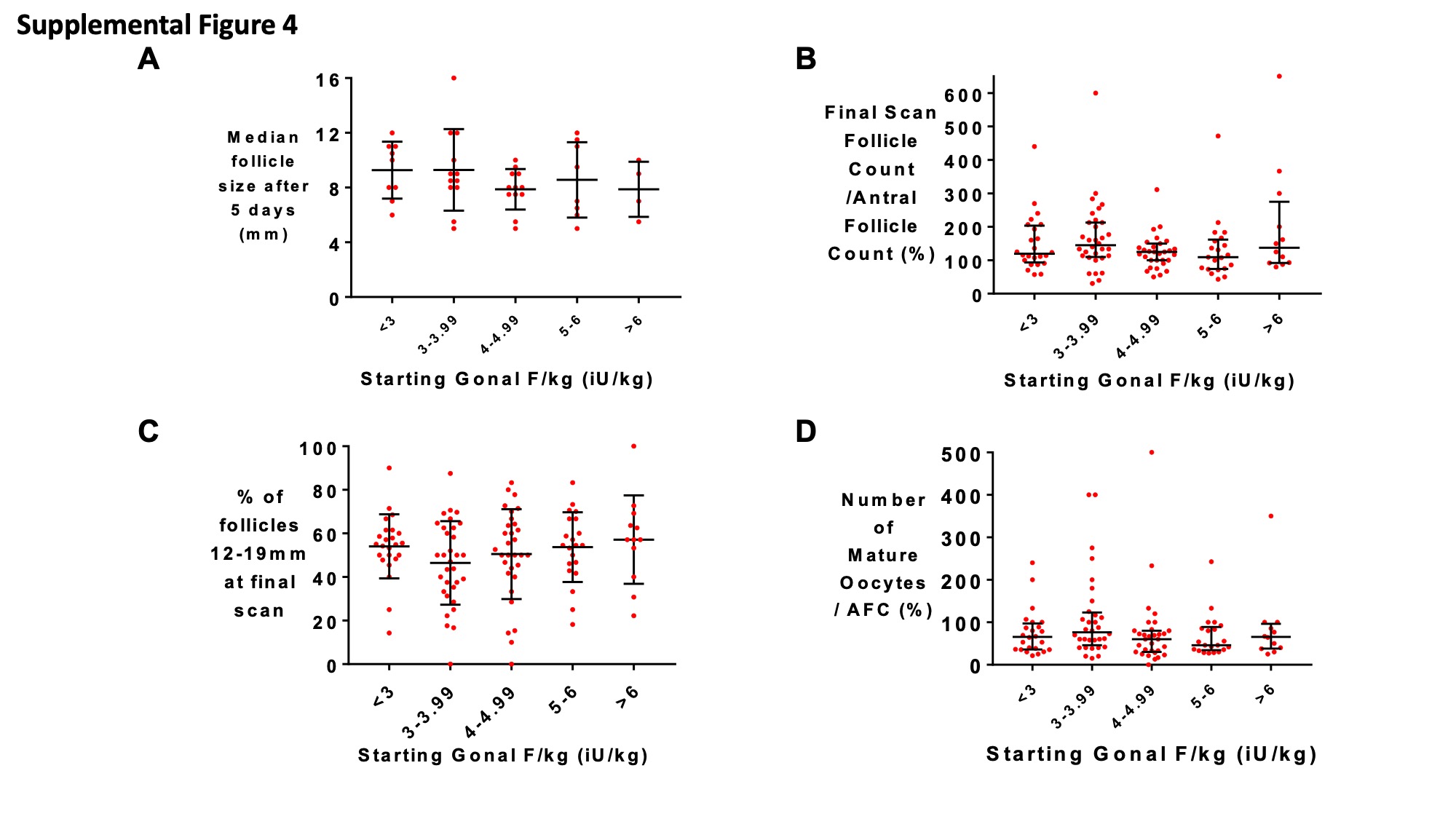

Supplement: Supplemental Figure 4 — Follicle growth in response to rFSH in a subset of predicted poorer responders (AFC≤15 and age≥35 years). (A) No significant differences were found between categories of starting rFSH dose (iU/kg) and median follicle size after 5 days (n = 45). Mean (±SD) is presented and categories were compared by one-way ANOVA with post hoc Tukey's multiple comparisons test. (B) No significant differences were observed between starting rFSH dose (iU/kg) and the proportion of the antral follicle count (AFC) recruited to grow (n = 120). Median (±IQR) is presented and categories were compared by the Kruskal-Wallis test with post hoc Dunn's multiple comparisons test. (C) No significant differences were observed between starting rFSH dose (iU/kg) and the proportion of 12–19 mm follicles at the end of stimulation (n = 120). Mean (±SD) is presented and categories were compared by one-way ANOVA with post hoc Tukey's multiple comparisons test. (D) No significant differences were observed between starting rFSH dose (iU/kg) and the proportion of antral follicles that yield a mature oocyte (n = 120). Median (±IQR) is presented and categories were compared by the Kruskal-Wallis test with post hoc Dunn's multiple comparisons test. [file Image_4.JPEG]

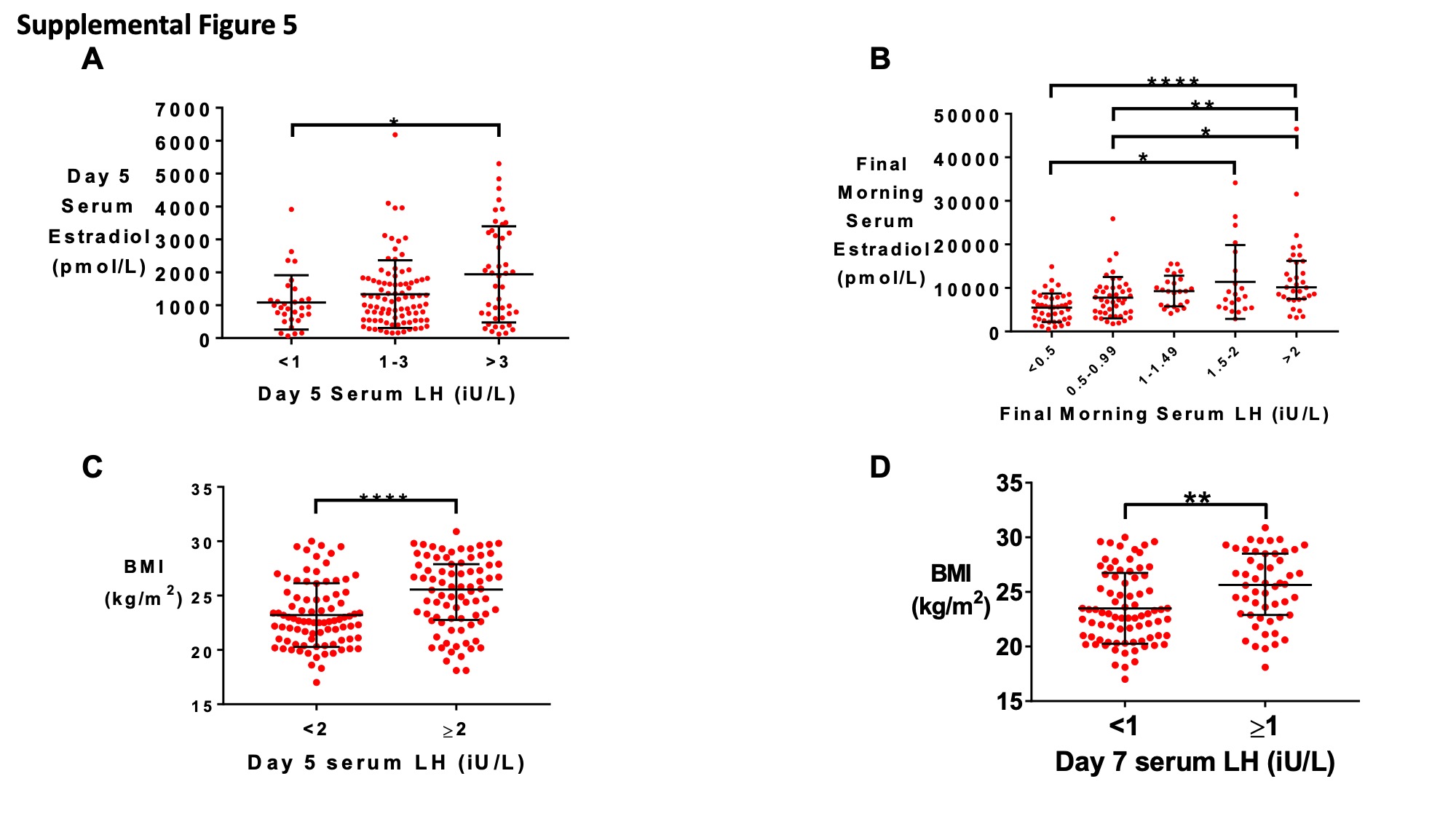

Supplement: Supplemental Figure 5 — (A,B) Both serum LH after 5 days of rFSH treatment (A; n = 166) and serum LH on the final morning before oocyte maturation trigger administration (B; n = 164) are positively associated with serum estradiol after 5 days of rFSH treatment. Median (±IQR) is presented and categories were compared by the Kruskal-Wallis test with post hoc Dunn's multiple comparisons test (n = 166). (C,D) A lower body mass index (BMI) is associated with lower serum LH levels both before (C; n = 167) and after (D; n = 131) administration of GnRH antagonist. *p < 0.05, **p < 0.01, ****p < 0.0001. Median (±IQR) is presented and categories were compared by the Mann Whitney U test (n = 167). [file Image_5.JPEG]
